# Efficacy of Oral Furosemide Test for Primary Aldosteronism Diagnosis

**DOI:** 10.1210/jendso/bvad147

**Published:** 2023-11-24

**Authors:** Thais C Freitas, Ana Alice W Maciel, Gustavo F C Fagundes, Janaina Petenuci, Lucas S Santana, Augusto G Guimaraes, Felipe Freitas-Castro, Victor Srougi, Fabio Y Tanno, Jose L Chambo, Maria Adelaide A Pereira, Luciana P Brito, Andrea Pio-Abreu, Luiz A Bortolotto, Ana Claudia Latronico, Maria Candida B V Fragoso, Luciano F Drager, Berenice B Mendonca, Madson Q Almeida

**Affiliations:** Divisão de Endocrinologia e Metabologia, Hospital das Clínicas, Unidade de Adrenal, Laboratório de Endocrinologia Molecular e Celular LIM/25, Faculdade de Medicina da Universidade de São Paulo, São Paulo, 05403-000, Brazil; Divisão de Endocrinologia e Metabologia, Hospital das Clínicas, Unidade de Adrenal, Laboratório de Endocrinologia Molecular e Celular LIM/25, Faculdade de Medicina da Universidade de São Paulo, São Paulo, 05403-000, Brazil; Divisão de Endocrinologia e Metabologia, Hospital das Clínicas, Unidade de Adrenal, Laboratório de Endocrinologia Molecular e Celular LIM/25, Faculdade de Medicina da Universidade de São Paulo, São Paulo, 05403-000, Brazil; Divisão de Endocrinologia e Metabologia, Hospital das Clínicas, Unidade de Adrenal, Laboratório de Endocrinologia Molecular e Celular LIM/25, Faculdade de Medicina da Universidade de São Paulo, São Paulo, 05403-000, Brazil; Divisão de Endocrinologia e Metabologia, Hospital das Clínicas, Unidade de Adrenal, Laboratório de Endocrinologia Molecular e Celular LIM/25, Faculdade de Medicina da Universidade de São Paulo, São Paulo, 05403-000, Brazil; Divisão de Endocrinologia e Metabologia, Hospital das Clínicas, Unidade de Adrenal, Laboratório de Endocrinologia Molecular e Celular LIM/25, Faculdade de Medicina da Universidade de São Paulo, São Paulo, 05403-000, Brazil; Divisão de Endocrinologia e Metabologia, Hospital das Clínicas, Unidade de Adrenal, Laboratório de Endocrinologia Molecular e Celular LIM/25, Faculdade de Medicina da Universidade de São Paulo, São Paulo, 05403-000, Brazil; Divisão de Urologia, Hospital das Clínicas, Faculdade de Medicina da Universidade de São Paulo, São Paulo, 05403-000, Brazil; Divisão de Urologia, Hospital das Clínicas, Faculdade de Medicina da Universidade de São Paulo, São Paulo, 05403-000, Brazil; Divisão de Urologia, Hospital das Clínicas, Faculdade de Medicina da Universidade de São Paulo, São Paulo, 05403-000, Brazil; Divisão de Endocrinologia e Metabologia, Hospital das Clínicas, Unidade de Adrenal, Laboratório de Endocrinologia Molecular e Celular LIM/25, Faculdade de Medicina da Universidade de São Paulo, São Paulo, 05403-000, Brazil; Divisão de Endocrinologia e Metabologia, Hospital das Clínicas, Unidade de Adrenal, Laboratório de Endocrinologia Molecular e Celular LIM/25, Faculdade de Medicina da Universidade de São Paulo, São Paulo, 05403-000, Brazil; Unidade de Hipertensão, Disciplina de Nefrologia, Hospital das Clínicas, Faculdade de Medicina da Universidade de São Paulo, São Paulo, 05403-000, Brazil; Unidade de Hipertensão, Instituto do Coração (InCor), Faculdade de Medicna da Universidade de São Paulo, São Paulo, 05403-900, Brazil; Divisão de Endocrinologia e Metabologia, Hospital das Clínicas, Unidade de Adrenal, Laboratório de Endocrinologia Molecular e Celular LIM/25, Faculdade de Medicina da Universidade de São Paulo, São Paulo, 05403-000, Brazil; Divisão de Endocrinologia e Metabologia, Laboratório de Hormônios e Genética Molecular LIM/42, Hospital das Clínicas, Faculdade de Medicina da Universidade de São Paulo, São Paulo, 05403-000, Brazil; Divisão de Oncologia Endócrina, Instituto do Câncer do Estado de São Paulo (ICESP), Faculdade de Medicina da Universidade de São Paulo, São Paulo, 01246-000, Brazil; Unidade de Hipertensão, Disciplina de Nefrologia, Hospital das Clínicas, Faculdade de Medicina da Universidade de São Paulo, São Paulo, 05403-000, Brazil; Unidade de Hipertensão, Instituto do Coração (InCor), Faculdade de Medicna da Universidade de São Paulo, São Paulo, 05403-900, Brazil; Divisão de Endocrinologia e Metabologia, Laboratório de Hormônios e Genética Molecular LIM/42, Hospital das Clínicas, Faculdade de Medicina da Universidade de São Paulo, São Paulo, 05403-000, Brazil; Divisão de Endocrinologia e Metabologia, Hospital das Clínicas, Unidade de Adrenal, Laboratório de Endocrinologia Molecular e Celular LIM/25, Faculdade de Medicina da Universidade de São Paulo, São Paulo, 05403-000, Brazil; Divisão de Oncologia Endócrina, Instituto do Câncer do Estado de São Paulo (ICESP), Faculdade de Medicina da Universidade de São Paulo, São Paulo, 01246-000, Brazil

**Keywords:** primary aldosteronism, diagnosis, furosemide, confirmatory test

## Abstract

**Context:**

Confirmatory tests represent a fundamental step in primary aldosteronism (PA) diagnosis, but they are laborious and often require a hospital environment due to the risks involved.

**Objective:**

To evaluate the efficacy of oral furosemide as a new confirmatory test for PA diagnosis.

**Methods:**

We prospectively evaluated the diagnostic performance of 80 mg of oral furosemide in 64 patients with PA and 22 with primary hypertension (controls). Direct renin concentration (DRC) was measured before, and 2 hours and 3 hours after the oral furosemide. In addition, the oral furosemide test was compared with 2 other confirmatory tests: the furosemide upright test (FUT) and saline infusion test (SIT) or captopril challenge test (CCT) in all patients with PA.

**Results:**

The cut-off of 7.6 µU/mL for DRC at 2 hours after oral furosemide had a sensitivity of 92%, specificity of 82%, and accuracy of 90% for PA diagnosis. In 5 out of 6 controls with low-renin hypertension, which might represent a PA spectrum, renin remained suppressed. Excluding these 6 controls with low-renin hypertension, the DRC cut-off of 10 µU/mL at 2 hours after oral furosemide had a sensitivity of 95.3%, specificity of 93.7% and accuracy of 95% for PA diagnosis. DRC after 3 hours of oral furosemide did not improve diagnostic performance. Using the cut-off of 10 µU/mL, the oral furosemide test and the FUT were concordant in 62 out of 64 (97%) patients with PA. Only 4 out of 64 cases with PA (6.4%) ended the oral furosemide test with potassium <3.5 mEq/L. Hypotension was not evidenced in any patient with PA during the test.

**Conclusion:**

The oral furosemide test was safe, well-tolerated and represents an effective strategy for PA investigation.

Primary hyperaldosteronism (PA) is characterized by autonomous aldosterone production leading to arterial hypertension, increased potassium excretion with variable degrees of hypokalemia, and cardiovascular damage [1, 2]. The prevalence of PA ranges from 5% to 10% of patients with arterial hypertension, reaching around 20% in resistant hypertension [3, 4]. PA is a very prevalent disease with a specific treatment (either surgical or pharmacological with mineralocorticoid antagonists), but PA diagnosis remains challenging due to its multiple steps (screening, confirmation, and determination of lateralization) [1].

Before proceeding to the determination of aldosterone lateralization by adrenal computed tomography (CT) and/or adrenal vein sampling (AVS), patients with hypertension with a positive screening for PA should undergo at least 1 confirmatory test [5]. The exception includes patients with spontaneous hypokalemia, suppressed renin levels plus aldosterone >20 ng/dL (554 pmol/L) [5]. There are different dynamic confirmatory tests for demonstrating aldosterone autonomous secretion based on aldosterone suppression or renin increase after a physiological stimulus [6]. However, a gold standard test has not been recommended by the main PA consensus [1, 7].

The most common confirmatory tests are the saline infusion test (SIT), the fludrocortisone suppression test (FST), and the captopril challenge test (CCT) [6]. The FST is considered the most effective test to suppress the renin–angiotensin–aldosterone system, but it consists of a high daily fludrocortisone dose (100 μg every 6 hours) for 4 days and demands inpatient care with intensive blood pressure and potassium monitoring [5]. The SIT, the most widely used confirmatory test, is associated with a sensitivity of 83% and a specificity of 75% when using the aldosterone level cut-off of 6.8 ng/dL at the recumbent position [8]. Ahmed et al [9] demonstrated that the seated SIT had a higher positivity rate than the recumbent SIT. Although sodium loading is the most used strategy for confirmatory tests, it should be avoided in patients with uncontrolled resistant hypertension, and heart and kidney failure [6]. On the other hand, the CCT is safer than sodium loading tests, but the criteria used for its interpretation are very variable and it has been associated with equivocal results [10, 11].

The furosemide upright test (FUT) evaluates the increase in renin after an acute furosemide injection, a response that is lacking in PA [12, 13]. A positive FUT for PA is defined by a plasma renin activity (PRA) below 2 ng/mL/hour after 2 hours in the upright position of an intravenous dose of 40 mg of furosemide [14]. Nanba et al [15]. showed that the FUT had a true positive rate of 88% compared with 60% for SIT in 57 Japanese patients with PA that underwent both tests. Despite this effective accuracy for PA diagnosis, the FUT has not been validated using direct renin concentration (DRC). The SIT had a lower accuracy in Japanese patients than what previously described in Chinese and Caucasian studies, suggesting an influence of ethnicity in the performance of confirmatory tests [15-17].

The PA case confirmation by a dynamic test still represents a very challenging step in PA work-up. The available confirmatory tests should be performed in an inpatient or outpatient facility under medical supervision, increasing the complexity of investigation and costs [6]. Considering the high prevalence of PA and the aforementioned drawbacks of the existing tests, it is essential to develop new strategies to confirm PA diagnosis after a positive screening. In 1972, Wallach et al [18] showed the efficacy of 60 mg of oral furosemide, as a single outpatient procedure to stimulate renin after 5 hours, in discriminating patients with essential hypertension from those with PA or renovascular hypertension. However, this approach has not been revisited so far.

In this prospective study, we evaluated the diagnostic performance of oral furosemide as a confirmatory test for PA in a cohort of patients with hypertension. In addition, the oral furosemide test was compared with 2 other confirmatory tests, the FUT and SIT or CCT (when sodium overload was contraindicated), in patients with PA.

## Materials and Methods

The study was approved by the Ethics Committees of the Hospital das Clinicas, University of São Paulo Medical School (#3.491.819) and written informed consent was obtained from all patients. We prospectively evaluated the diagnostic efficacy of oral furosemide in 64 consecutive patients with diagnosis of PA referred to the Adrenal Unit from 2019 to 2021 (Fig. S1 [19]). In addition, the oral furosemide test was performed in 22 individuals with primary hypertension as a control group (Fig. S2 [19]). The estimated sample size of 46 cases and 23 controls (2:1 ratio) was determined to achieve a study power of 80%. The exclusion criteria were as follows: (1) patients with glomerular filtration rate (Chronic Kidney Disease Epidemiology Collaboration) < 60 mL/min because we cannot predict the response to furosemide due to renal impairment; and (2) hypokalemia (<3.5 mEq/L) under maximum tolerated dose of oral potassium supplementation due to the risk of worsening hypokalemia.

Clinical, biochemical, and imaging data were collected from patient records. The algorithm for PA investigation followed the 2016 Endocrine Society Guideline for PA management [1]. In our institution, a positive screening for PA was defined as an aldosterone/direct renin concentration (A/DRC) ratio >2 ng/dL/µU/mL with concomitant aldosterone levels >8 ng/dL (277 pmol/L). An A/DRC ratio >2 ng/dL/µU/mL has a sensitivity of 92% for PA diagnosis when measured by chemiluminescent immunoassays [20].

All patients with PA, even those patients with spontaneous hypokalemia, suppressed renin levels plus aldosterone concentrations >20 ng/dL (554 pmol/L), underwent 2 confirmatory tests (Fig. S1): (1) FUT and (2) SIT or CCT (when sodium overload was contraindicated). After PA diagnosis was confirmed by 2 positive confirmatory tests, the patients underwent the oral furosemide test in order to evaluate its efficacy. The FUT was performed in the PA cohort to compare the effect of intravenous vs oral furosemide to stimulate renin concentration in patients with PA and to validate this test using DRC (instead of PRA). The gold standard criteria for PA diagnosis were AVS lateralization and/or biochemical cure for PA after unilateral adrenalectomy, and 2 positive confirmatory tests.

Regarding the group with primary hypertension, all cases had a negative PA screening after removing interfering antihypertensive medications. Therefore, we performed the oral furosemide test in this group to evaluate the effect of oral furosemide in increasing renin levels in a group clearly without PA.

### Confirmatory Test Protocols

All confirmatory tests were performed between 7 and 9 Am after an overnight fast. The confirmatory tests were performed at least 7 days apart. At the end of SIT, an aldosterone concentration >8.8 ng/dL (243 pmol/L) measured by immunoassay was used to define a positive test [21]. This aldosterone cut-off of 8.8 ng/dL is higher than the previously proposed threshold of 6.1 ng/dL for the seated SIT, but it was recently associated with a decrease in the false positive rate due to immunoassay inaccuracy when compared with mass spectrometry [9, 21]. The CCT was considered positive for PA diagnosis if aldosterone remained >12 ng/dL (330 pmol/L) or aldosterone suppression was <30% after 2 hours of 50 mg of oral captopril [1]. For the FUT, DRC was determined after 2 hours of an intravenous injection of 40 mg of furosemide. A positive FUT was defined as PRA < 2 ng/mL/hour (DRC < 24 µU/mL considering the conversion factor of 12) [15].

The protocol for the oral furosemide test consisted in the administration of 80 mg of furosemide. The bioavailability of oral furosemide varies from 47% to 64% [22]. Therefore, 80 mg of oral furosemide is expected to have a bioavailability similar to an intravenous 40-mg dose. DRC, aldosterone, and potassium levels were measured before, and 2 hours and 3 hours after the oral furosemide dose. Blood pressure and heart rate were determined before and at the end of the test. Diuresis induced by furosemide reaches a maximum at 2 to 3 hours and lasts for about 4 hours [23, 24].

Spironolactone was suspended at least 4 weeks before PA screening. Patients taking spironolactone before the first PA screening were included only if DRC was suppressed (<4 µU/mL) with aldosterone >15 ng/dL (415 pmol/L) and spironolactone discontinuation was not possible (ie, refractory hypertension or very severe hypokalemia with history of arrhythmia or cardiac arrest).

For patients with aldosterone levels ≤15 ng/dL (415 pmol/L) or unsuppressed DRC (>10 µU/mL), spironolactone and diuretics were stopped and other antihypertensive medications were modified to hydralazine, calcium channel blockers, and/or doxazosin whenever possible. Beta-blockers were changed for verapamil, unless in the presence of coronary heart disease.

### Aldosterone and Renin Assays

Aldosterone and DRC were measured using an automated chemiluminescence-based assay (LIAISON kit, DiaSorin, Salugia, Italy) in all patients. Aldosterone concentration was measured in serum and DRC in plasma with ethylenediaminetetraacetic acid. Functional sensitivity (lowest concentration at which the analyte can be reliably detected) was 3 ng/dL (83.2 pmol/L) for aldosterone and 4 µU/mL for DRC. DRC normal range in seated position varies from 4.6 to 46 µU/mL. The interassay coefficient of variation for the aldosterone assay ranged from 12% at lower concentrations to 6% at higher concentrations. The interassay coefficient of variation for the renin assay was 5.5%.

### Definition of Lateralization and Follow-up

Sequential AVS was performed under cosyntropin continuous infusion by an experienced interventional radiologist. Successful catheterization was defined by a selectivity index ≥5. Unilateral disease was defined by a lateralization index ≥4 [25, 26]. Bilateral PA was defined by bilateral aldosterone excess in AVS. Unilateral disease was determined by biochemical PA cure after unilateral adrenalectomy. Undetermined lateralization included patients that did not undergo AVS and/or not present biochemical PA cure after unilateral adrenalectomy.

Clinical and biochemical success after adrenalectomy for unilateral disease was evaluated according to the Primary Aldosteronism Surgical Outcome (PASO) criteria [27, 28]. Biochemical cure was defined as correction of hypokalemia and normalization of the aldosterone to renin ratio after 6 months of surgery. In patients with a raised aldosterone to renin ratio postsurgery, aldosterone secretion should be suppressed in a confirmatory test [28]. Complete clinical success was defined as a blood pressure <140 × 90 mmHg without antihypertensive drugs after 6 months of follow-up. The cut-off of blood pressure ≥140 × 90 mmHg was used to define stage 1 hypertension in both European and Brazilian guidelines for the management of arterial hypertension [29, 30].

### Statistical Analysis

The estimated sample size of 46 cases and 23 controls (2:1 ratio) was determined to achieve a study power of 80%, assuming a type 1 error (alpha) of 0.05 and an area under the receiver operating characteristic (ROC) curve of 0.7 (MedCalc Software Ltd, Version 20.009, Ostend, Belgium). ROC curves have also been applied to assess the sensitivity and specificity of the renin levels at 2 hours and 3 hours of the furosemide oral test.

Data analysis was performed using SPSS Software (25.0; SPSS Inc., Chicago, IL, USA). Initially, a descriptive analysis of the data was presented as absolute (n) and relative (%) frequencies for qualitative variable. The main summary measures, such as measures of position and dispersion, were provided for the quantitative variables. Fisher's exact test or the chi-square test was applied to assess a possible association of qualitative variables in relation to the outcomes of interest. The comparison of data from quantitative variables in relation to the group variable was performed using the nonparametric Mann–Whitney U test or the parametric Student's t test. The Shapiro–Wilk test was used to assess the normality of data in each of the groups. In case of violation of the normality assumption, the nonparametric test was applied. In addition, Spearman's correlation coefficient was calculated to determine the correlation between 2 continuous variables.

Subsequently, in order to assess the effect of time and group (PA or control group) on repeated measures (aldosterone, DRC, and potassium levels) during the oral furosemide test, the generalized estimation equation (GEE) model was performed using R software version 4.0 (R Core Team, 2020). Due to the form of data distribution of aldosterone, DRC, and potassium, the Gamma distribution was considered assuming a log binding function. Furthermore, we specified the unstructured structure for the working correlation matrix to quantify the correlation between the observations of the same group, adjusted by the covariates present in the model. Estimated marginal means were obtained from an adjusted linear regression model and Bonferroni's correction was performed to counteract the multiple comparisons. The significance level adopted was 5% in all analysis considering a bilateral test. Thus, results whose *P* values are less than .05 are considered to be statistically significant.

## Results

### Study Population Characteristics

Baseline information of the study population is presented in Table 1. patients with PA had a median age at the diagnosis of hypertension of 38 years (range, 14-64), which was significantly lower than controls with hypertension without PA (57 years, range 21-75; *P* < .0001). The criteria for PA screening in both groups are listed in Table 1. Among the 22 patients without PA (control group), 6 patients had low-renin hypertension with suppressed DRC (<4 µU/mL) and low aldosterone levels (<7 ng/dL; 194 pmol/L). PA screening was repeated in these cases and aldosterone concentration remained <7 ng/dL.

**Table 1.** Clinical and biochemical characteristics of hypertensive patients with and without primary aldosteronism diagnosis

	PA n = 64	Primary hypertension*^b^* n = 22	*P*
Female sex n (%)	41 (64)	17 (77.3)	.254
Age at diagnosis of hypertension (years)*^a^*	38 (14-64)	57 (21-75)	<.0001
Age at PA diagnosis	51 (23-76)		
≥3 Drugs for hypertension treatment n (%)	44 (68.8)	6 (27.3)	.001
Aldosterone (ng/dL)*^a^*	19.9 (7.3-217)	7.1 (3.5-27.7)	<.0001
DRC (µUI/mL)*^a^*	4.0 (4.0-8.8)	8.9 (4.0-147)	<.0001
A/DRC ratio*^a^*	4.6 (1.8-36)	1.0 (0.1-2.2)	<.0001
Criteria for PA screening			
Sustained blood pressure >150 × 100 mmHg	64 (100)	12 (54.5)	<.0001
Resistant hypertension	43 (67.2)	5 (22.7)	.001
Hypokalemia and hypertension	35 (54.7)	1 (4.5)	<.0001
Adrenal incidentaloma and hypertension	4 (18.2)	3 (13.6)	.274
Family history of early onset hypertension or cerebrovascular accident <40 years	21 (32.8)	2 (9.1)	.017
Sleep obstructive apnea and hypertension	6 (9.4)	5 (22.7)	<.0001

Abbreviations: A, aldosterone; DRC, direct renin concentration; PA, primary aldosteronism.

^*a*^Median (range).

^*b*^Six patients presented suppressed renin (<4 µUI/mL) and low aldosterone levels (<7 ng/dL; 194 pmol/L) characterizing low renin hypertension. For these patients, the PA screening was negative.

Almost 70% of patients with PA had resistant hypertension were taking more than 3 antihypertensive medications. Hypokalemia was evidenced in 35 out of 64 PA cases (54.7%). PA screening was repeated for the cases with the first aldosterone measure <10 ng/dL. All 64 patients with PA underwent the oral furosemide test, the FUT, and a third confirmatory test (50 SIT and 14 CCT).

AVS success rate was 88.6% (39 out of 44 procedures). Regarding the lateralization of aldosterone excess, 26 cases (40.6%) had bilateral disease and 22 cases (34.4%) unilateral disease. PA lateralization was not determined in 16 cases (25%) (Fig. S1 [19]). Among the patients who underwent unilateral adrenalectomy, 22 out of 24 cases (91.6%) had complete biochemical success and 10 out of 24 cases (41.6%) had complete clinical success.

### Oral Furosemide Test

**Figure 1.**
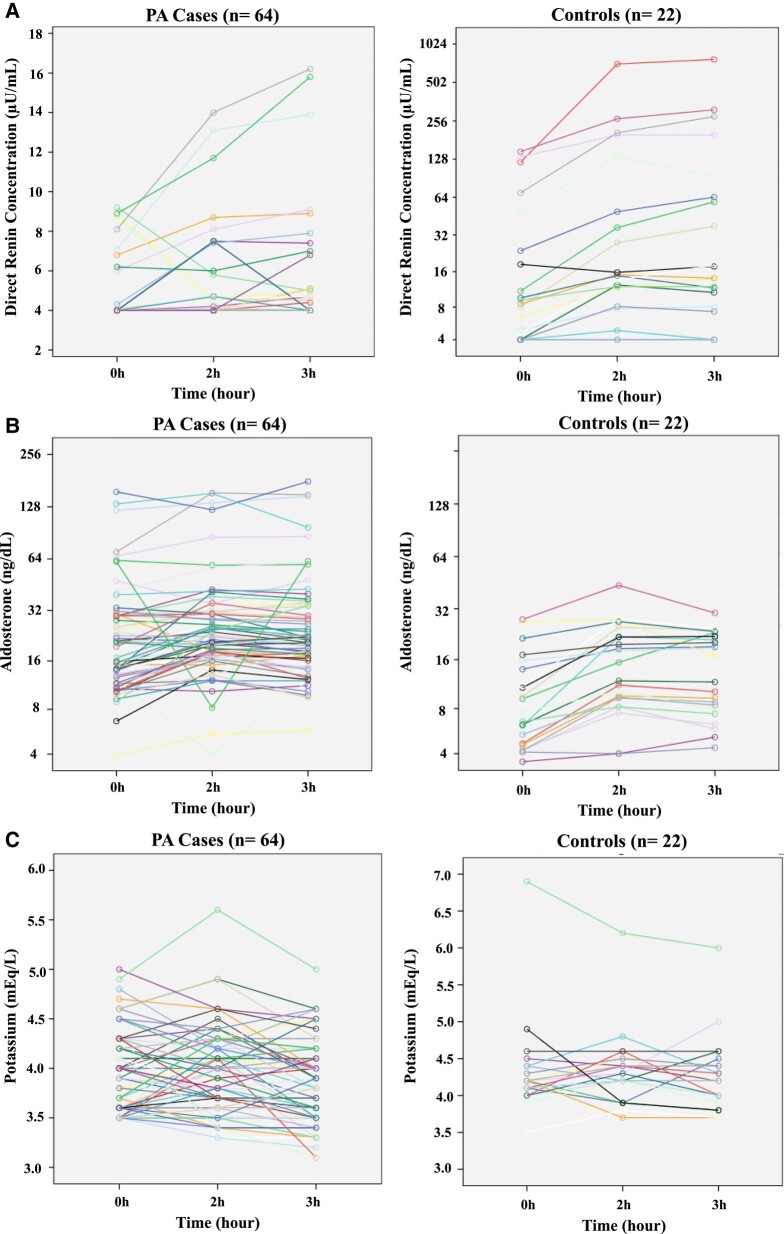
Repeated measures (basal, 2 hours and 3 hours after 80 mg of oral furosemide) of direct renin concentration (A), aldosterone (B), and potassium (C) levels in the group of cases (patients with PA) and controls (primary hypertension and low-renin hypertension). PA, primary aldosteronism. Renin remained suppressed at baseline and during the test in several cases (for this reason the lines are overlapping).

**Figure 2.**
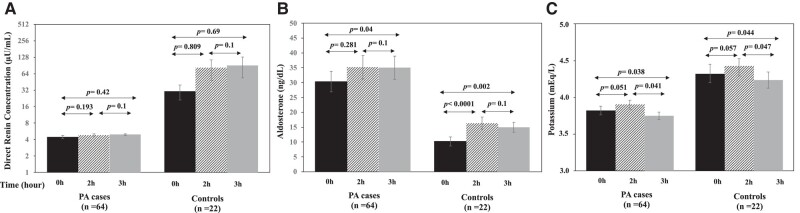
Estimated marginal means (± SE) of direct renin concentration (A), aldosterone (B) and potassium (C) during different times of the oral furosemide test in the PA and control groups. Bonferroni's correction was performed to counteract the multiple comparisons. SE, standard error; PA, primary aldosteronism.

During the oral furosemide test, concentrations of aldosterone, DRC and potassium at basal condition and after 2 hours and 3 hours are showed for all cases (PA and control group) in Fig. 1. The repeated measures of aldosterone, DRC, and potassium were analyzed by the GEE model to assess the effect of the time and the group (case and control). The GEE model showed that there was a significant interaction between group and time (Table 2). DRC was significantly lower in the PA group (case) than in controls and increased with the time. After Bonferroni's correction for multiple comparisons, basal DRC at the case group (4.53 ± 0.17 µU/mL) was not significantly different than DRC at 2 hours (4.88 ± 0.27 µU/mL, *P* = .193) and at 3 hours (5.03 ± 0.32 µU/mL, *P* = .42). Although the GEE model showed a significant effect of time for DRC, this effect was not demonstrated in the control group after multiple comparisons because of the high degree of variability in DRC during the test (Fig. 2A).

**Table 2.** Effect of group (case and control) and time on repeated measures of aldosterone, potassium and direct renin concentrations during the oral furosemide test using the multivariate generalized estimating equation

Variable	Category	Coefficient (B)	SE	Exp (B)	95% CI	*P*
**Aldosterone concentration**						
Intercept		2.32	0.14	10.22	7.63-13.7	<.001
Group	Case (PA)	1.08	0.18	2.97	2.05-4.29	<.001
	Control	Ref		1		
Time	3 hours	0.38	0.1	1.46	1.18-1.79	<.001
	2 hours	0.47	0.08	1.6	1.37-1.87	<.001
	Basal	Ref		1		
**Potassium**						
Intercept		1.46	0.03	4.32	4.06-4.59	<.001
Group	Case (PA)	−0.12	0.03	0.88	0.83-0.94	<.001
	Control	Ref		1		
Time	3 hours	−0.02	0.01	0.98	0.96-1.0	.049
	2 hours	0.02	0.01	1.02	0.99-1.048	.125
	Basal	Ref		1		
**Direct renin concentration**						
Intercept		3.41	0.31	30.51	16.68-55.79	<.001
Group	Case (PA)	−1.91	0.31	0.14	0.08-0.27	<.001
	Control	Ref		1		
Time	3 hours	1.09	0.25	2.98	1.82-4.87	<.001
	2 hours	0.98	0.24	2.67	1.65-4.34	<.001
	Basal	Ref		1		

Abbreviations: PA, primary aldosteronism; Ref, category of reference.

A significant effect of time and group was also demonstrated for aldosterone concentration (Table 2). In the PA group, aldosterone concentration significantly increased at 3 hours compared with the basal level (32.31 ± 4.13 ng/dL vs 27.87 ± 3.61 ng/dL, respectively; *P* = .04), but this effect was higher in the control group (Fig. 2B). In controls, aldosterone levels increased significantly at 2 hours (16.37 ± 2.09 ng/dL) and at 3 hours (14.95 ± 1.69 ng/dL) when compared with the basal level (10.23 ± 1.56 ng/dL; *P <* .0001 and *P* = .002, respectively).

Potassium levels were lower in the PA group than in the control group and slightly decreased at 3 hours (2% in medium levels) (Table 2). In patients with PA, potassium levels decreased at 3 hours (3.82 ± 0.05 mEq/L) compared with basal (3.93 ± 0.05 mEq/L, *P* = .038) and 2 hours (3.98 ± 0.05 mEq/L, *P* = .041). Similarly, potassium levels decreased at 3 hours (4.26 ± 0.1 mEq/L) compared with basal (4.33 ± 0.14 mEq/L, *P* = .044) and 2 hours (4.33 ± 0.1 mEq/L, *P* = .047) in the control group (Fig. 2C). Among the patients with PA, only 2 (3.1%) cases at 2 hours and 4 out of 64 cases (6.2%) at 3 hours had potassium levels between 3.0 and 3.5 mEq/L. Three cases ended the test with potassium levels >5.0 mEq/L. Since these 3 cases had normal kidney function, hyperkalemia was probably caused by hemolysis.

### Sensitivity and Specificity of the Oral Furosemide Test

**Figure 3.**
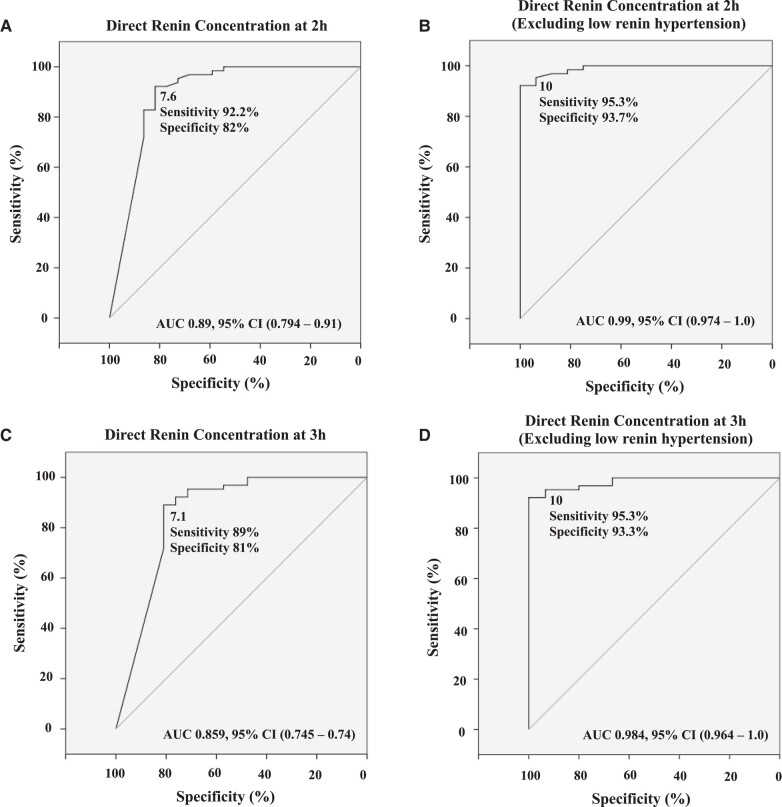
Receiver operating characteristic (ROC) curves showing the optimal cut-offs for direct renin concentration at 2 hours (A and B) and at 3 hours (C and D) of oral furosemide test for PA diagnosis. Area under the curves (AUC) are provided and optimized cut-offs were assessed according to Youden indexes with associated diagnostic sensitivities and specificities. Since low-renin hypertension is considered a spectrum of PA, we re-analyzed the data excluding these patients from the control group (B and D). PA, primary aldosteronism.

ROC curve showed the optimal cut-off of 7.6 µU/mL for DRC at 2 hours during the oral furosemide test with a sensitivity of 92% and a specificity of 82% for PA diagnosis (Fig. 3A). The predictive positive and negative values were 93.7% and 78.3%, respectively. The diagnostic accuracy for the 7.6 µU/mL cut-off at 2 hours was 90%. At 3 hours of the oral furosemide test, the cut-off of 7.1 µU/mL for DRC had a sensitivity of 89% and specificity of 81% for PA diagnosis (Fig. 3C).

Six out of 22 hypertensive patients in the control group had low-renin hypertension (suppressed DRC and low aldosterone levels after repeated measures). Among these 6 cases, 4 remained with DRC < 7.6 µU/mL at 2 hours and < 7.1 µU/mL at 3 hours (Fig. S3 [19]). Five out of these 6 cases had DRC < 10 µU/mL at 2 hours. The cut-off of 10 µU/mL at 2 hours had a higher sensitivity of 95.3%, but a lower specificity of 73%. Since low-renin hypertension has been recently considered a spectrum of aldosterone autonomy [31], we reanalyzed the data excluding the cases with low-renin hypertension from controls (Fig. 3B and 3D). In this scenario, the DRC cut-off of 10 µU/mL at 2 hours had a sensitivity of 95.3% and specificity of 93.7% for PA diagnosis. Using the cut-off of 10 µU/mL at 2 hours, the predictive positive and negative values were 98.4% and 83.3%, respectively. The diagnostic accuracy for the 10 µU/mL at 2 hours cut-off was 95%. The cut-off of 10 µU/mL at 3 hours offered the same diagnostic performance as demonstrated at 2 hours.

### Comparison With the FUT and Other Confirmatory Tests

**Figure 4.**
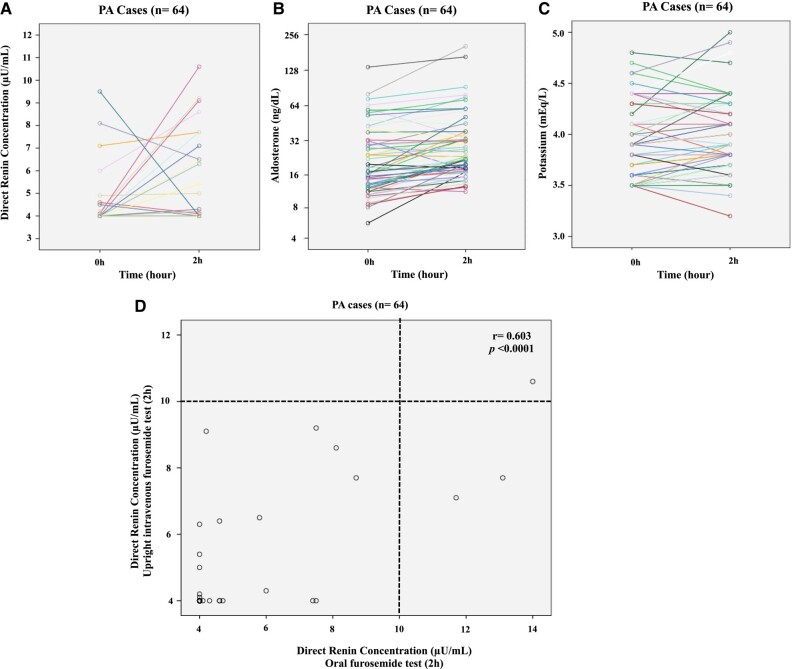
Repeated measures (basal and 2 hours after intravenous 40 mg furosemide) of direct renin concentration (A), aldosterone (B), and potassium (C) levels in 64 patients with PA during the intravenous furosemide upright test (FUT). (D) Direct renin concentration (DRC) at 2 hours of the oral furosemide test significantly correlated with the DRC at 2 hours of the FUT. Using the cut-off of 10 µU/mL, oral and intravenous furosemide test were concordant in 62 out of 64 (97%) of patients with PA. PA, primary aldosteronism. Renin remained suppressed at baseline and during the test in several cases (for this reason the lines are overlapping).

In order to compare the effect of oral and intravenous furosemide in DRC, all 64 patients with PA also performed the FUT (Fig. 4A-4C). DRC did not increase significantly in patients with PA after 2 hours of intravenous furosemide injection when compared to basal condition (4.67 ± 0.19 µU/mL vs 4.28 ± 0.12 µU/mL, respectively; *P* = .058). Aldosterone levels increased significantly after 2 hours of the FUT when compared with basal levels (32.8 ± 4.13 ng/dL vs 26.25 ± 2.78 ng/dL, respectively; *P* < .0001). Unexpectedly, potassium levels did not decrease at the end of the FUT. Considering the previous proposed cut-off of 2 ng/mL/h (DRC = 24 µU/mL using the conversion factor of 12), FUT was positive for PA diagnosis in all patients with PA. However, a renin cut-off using DRC has not been previously established during the FUT. In our cohort, the 97.5th percentile point for DRC was 9.8 µU/mL at 2 hours of the FUT.

DRC at 2 hours of the oral furosemide test significantly correlated with DRC at 2 hours of the FUT (r = 0.603, *P <* .0001) (Fig. 4D). Using the cut-off of 10 µU/mL, the oral furosemide test and the FUT were concordant in 62 out of 64 (97%) patients with PA. Regarding the other confirmatory tests, the SIT was positive in 40 out of 50 (80%) patients with PA using the cut-off of 8.8 ng/dL. The CCT was positive in 12 out of 14 (85.7%) PA cases.

### Blood Pressure and Heart Rate During the Oral Furosemide Test

During the oral furosemide test, the systolic blood pressure reduced similarly in the case and control groups (Δ −11.8 ± 2.12 mmHg and −8.4 ± 3.6 mmHg, respectively; *P* = .81) (Fig. S4 [19]). The reduction in diastolic blood pressure was not significantly different in both groups (case −6.2 ± 1.55 mmHg vs control −0.6 ± 1.57 mmHg, *P* = .64). The increase in heart rate was similar in the case and control groups (Δ 8.2 ± 1.7 bpm and 14.7 ± 3.2 bpm, respectively; *P* = 0.476). Arterial hypotension was not observed at the end of the oral furosemide test in any patient of both groups.

## Discussion

In this study, we investigated the role of a novel confirmatory test for the diagnosis of PA. The oral furosemide test had an excellent diagnostic accuracy for PA. Moreover, the oral furosemide test was safe and well-tolerated. The prolongation of the oral furosemide test beyond 2 hours did not improve its diagnostic performance. Despite its high prevalence and associated complications, PA remains largely underdiagnosed, with less than 2% of risk groups tested [32]. The high complexity of PA work-up definitely contributes to this low screening rate. Several confirmatory tests have been proposed to confirm the PA diagnosis after a positive screening [6]. The FST demands inpatient care with intensive blood pressure and potassium monitoring, which is particularly complicated in patients with PA with resistant hypertension or severe hypokalemia. An inpatient or outpatient facility under medical supervision are also requested for other confirmatory tests (such as SIT, CCT, or FUT). Unfortunately, an outpatient facility for dynamic tests is not widely available in several countries, which becomes a very important limitation for PA investigation.

The SIT is the most employed confirmatory test for PA diagnosis [5]. Nevertheless, the SIT should be indicated very cautiously for patients with resistant hypertension, which represented the majority of patients with PA in our cohort. The diagnostic accuracy of SIT for PA diagnosis varies significantly across several cohorts depending on the test protocol and aldosterone cut-off [8, 9, 15-17]. In our study, the detection rate of the SIT was 80% among the PA cohort, which was inferior to the FUT and the oral furosemide test. In agreement with our finding, Nanba et al [15] previously demonstrated that the FUT had a higher diagnostic accuracy than SIT for PA diagnosis.

Very recently, Leung et al [33] performed a systematic review and meta-analysis of the performance of confirmatory tests for PA diagnosis. This meta-analysis included studies for recumbent SIT, seated SIT, salt loading test, FST, and CCT. There were large variations in how confirmatory tests were conducted and interpreted, resulting in an excess of missed cases in most situations [33]. Few studies evaluated SIT compared with another standard confirmatory test [16, 34, 35]. The FUT performance was not included in this meta-analysis. This systematic review concluded the indication of confirmatory tests are based on very low–quality evidence and their routine use should be reconsidered [33]. More recently, another meta-analysis evaluated the diagnostic accuracy of exclusion tests for PA and showed that CCT and SIT did not add a diagnostic gain over the aldosterone to renin ratio [36].

PA has been defined as a wide spectrum multidimensional disease, varying from subclinical forms to more florid PA [37-39]. There is growing evidence for inappropriate aldosterone production playing a role in a larger subset of patients with hypertension [40, 41]. Recently, Brown et al [31] showed that 24-hour urinary aldosterone levels following an oral sodium suppression test were continuously increased throughout hypertension categories, varying from 11% in normotension to 22% in resistant hypertension, suggesting a continuum of renin-independent aldosterone production in patients with hypertension [31]. Based on these findings, the distinction between “biochemically overt primary aldosteronism” and “renin-independent aldosterone production” is arbitrary. PA would be better defined as a syndrome of renin-independent aldosterone production [31, 37]. A suppressed renin level (not increased after oral furosemide stimulus) could be an earlier evidence of autonomous aldosterone production, because any measured aldosterone level (even <8-10 ng/dL) should be inappropriate if renin is suppressed.

Low-renin phenotype has been previously characterized in hypertension [42, 43]. In the PATHWAY-2 trial, patients with uncontrolled resistant hypertension were randomly assigned to sequential crossover treatment with spironolactone, bisoprolol, doxazosin, and placebo [44]. The efficacy of spironolactone was superior in patients with low-renin levels, in whom PA has been previously excluded [44]. This finding supports the concept of a spectrum of renin-independent aldosterone production. In our study, 6 hypertensive patients with suppressed DRC and low aldosterone levels were included. Therefore, we re-analyzed our data excluding these patients from the control group to determine the optimal cut-off for DRC at the oral furosemide test. Five out of 6 patients with low-renin hypertension remained with DRC <10 µU/mL at the end of the oral furosemide test. In this scenario, a confirmatory test evaluating the effect of furosemide in increasing renin levels would be more accurate to identify a less severe spectrum of PA, instead of evaluating aldosterone suppression.

The strength of this study includes the analysis of a novel confirmatory test compared with 2 other standard confirmatory tests in all patients with PA. In addition, the FUT was first evaluated using DRC instead of plasma renin activity. A third strength of our study was the investigation of the DRC response after the oral furosemide in low-renin hypertensive patients. One limitation was the use of interfering anti-hypertensive medications in patients with PA. Since most of patients with PA had severe hypertension, it is not possible in clinical practice to change all medications that can affect aldosterone/renin profile. However, it should be emphasized that patients with PA using interfering antihypertensive medications were included only if DRC was suppressed. It has been previously demonstrated that conclusive AVS lateralization is often achieved in patients with severe PA despite mineralocorticoid receptor antagonist use [45]. Another potential limitation is the fact that we did not investigate all consecutive hypertensive patients referred to our Institution, because changing antihypertensive medications was challenging in patients with resistant hypertension and low probability of PA (DRC > 30-50 µU/mL under interfering drugs).

We propose here a novel and effective confirmatory test that can be safely performed as an outpatient procedure. Patients under PA investigation (without hypokalemia or with normal potassium levels under replacement) can take furosemide 80 mg orally and go to a laboratory to collect a blood sample to DRC determination after 2 hours. This new approach might facilitate the PA investigation in centers without outpatient facilities for endocrine dynamic tests. In conclusion, the oral furosemide test was safe, well-tolerated and represents an effective strategy for PA investigation. This a proof of concept study that might trigger future studies to confirm the diagnostic value of the oral furosemide test.

## Data Availability

The data that support the findings of this study are available from the corresponding author upon reasonable request.

## Abbreviations

AVS: adrenal vein sampling
CCT: captopril challenge test
CT: computed tomography
DRC: direct renin concentration
FST: fludrocortisone suppression test
FUT: furosemide upright test
GEE: generalized estimation equation
PA: primary aldosteronism
PRA: plasma renin activity
ROC: receiver operating characteristic
SIT: saline infusion test
